# Sawing Force Prediction Model and Experimental Study on Vibration-Assisted Diamond Wire Sawing

**DOI:** 10.3390/mi13112026

**Published:** 2022-11-19

**Authors:** Chenpu Zhang, Zhikui Dong, Yanheng Zhao, Ziliang Liu, Shang Wu, Jiahao Yang

**Affiliations:** School of Mechanical Engineering, Yanshan University, No. 438 West Hebei Avenue, Qinhuangdao 066004, China

**Keywords:** diamond wire sawing, brittle materials, vibration assistance, sawing force, surface quality, multi-wire sawing

## Abstract

Diamond wire sawing is the main machining technology for slicing various brittle materials, such as crystalline silicon, SiC, and NdFeB. Due to their high hardness and high brittleness, as well as the ease with which the surfaces of machined materials are damaged, it is difficult to further improve the sawing efficiency and the surface quality based on research conducted on the original machining method. In this paper, a vibration-assisted diamond wire sawing method is proposed. We analyzed the impact of load on the ingot, motion trajectory, and sawing depth of the abrasive particles, and a macroscopic sawing force prediction model for the vibration-assisted sawing method was established and verified via experiments. Based on the single-wire-sawing experiment and prediction model, the influences of the vibration parameters and sawing parameters on the sawing force were determined. The influences of vibration assistance on the surface quality, including the roughness profile, waviness profile, thickness profile, Ra, and Rz, were explored through single-wire-sawing experiments, and the influences of vibration assistance on the geometric parameters of slices, such as the total thickness variation (TTV) and warp, were explored through multi-wire-sawing experiments. It was found that vibration-assisted sawing can reduce sawing force and improve surface quality.

## 1. Introduction

In recent years with the development of material science and industrial technologies, brittle materials, such as sapphire, NdFeB, and SiC, have been widely used in the electronic industry, photovoltaic industry, aerospace industry, and other fields. Fixed abrasive wire sawing has become the main machining method for sawing brittle materials due to its technical advantages, such as narrow incision, good surface quality, and strong sawing ability, which help it to achieve low total thickness variation (TTV), a smaller damage layer, and fewer surface cracks [1,2,3].

In the case of diamond wire saws, the existing processing efficiency and wafer quality are difficult to further improve by changing the process parameters. Therefore, some scholars have tried to optimize diamond wire sawing technology using vibration-assisted methods.

For conventional diamond wire sawing, scholars have explored the influence of the sawing parameters on the sawing force through experiments and have established corresponding mathematical models, which suggest that increasing the size of the abrasive particles, increasing the wire speed, and reducing the feed speed [4,5,6] may reduce the sawing force, reduce crack damage on the surface, and improve surface quality. Pala U [7] explored the effect of wire speed on the rate of change of the sawing force. Huang [8] found that there was a good linear relationship between the sawing force and material removal rates (MRRs). Wang PZ [9,10] analyzed sawing force in the process of abrasive scribing and studied the frictional force of abrasive particles and determined a sawing force model with respect to process parameters and wire parameters. Wu CH [11] studied the distribution laws of multiple abrasive particles and established a macro-mathematical model between sawing force and the three process parameters of wire speed, feed speed, and wire radius. Li SJ [12,13] established a sawing force model by analyzing the contact length during sawing.

In the process of establishing a cutting force model, it is necessary to analyze the mechanisms of crack damage and material removal between abrasive particles and the material surface. Important parameters, such as the force on abrasive grains, cutting depth, and undeformed chip thickness, are determined during the material removal process. Shengyao Yang [14] revealed the deformation and material removal mechanisms of KDP crystals by analyzing the frictional force and normal force in a nanoscratching process. Junqin Shi [15] investigated the plastic deformation and tribological properties of metal materials during mechanical polishing. However, the speeds employed in the experiment were from μm/s to mm/s, which is three to six orders of magnitude lower than the m/s used in machining. To overcome this challenge, Bo Wang [16] developed a novel approach involving single-particle cutting at speeds of m/s, and the nanoscale depth of the cut and the formation mechanism of the new phase were determined. Zhenyu Zhang [17] found that brittle materials failed under fracture due to the initiation and propagation of cracks. The force, the depth of the cut, and the size of the plastic deformation in single-grain grinding were calculated. This opened a new pathway to investigate the mechanisms of abrasive machining. Moreover, a novel model of maximum undeformed chip thickness was suggested for multi-abrasive particles, and a balance between the rate of material removal and the total volume of nanoparticles generated per unit time was proposed [18]. These studies have made great contributions to traditional abrasive machining methods, such as polishing, grinding, and sawing. Zhenyu Zhang and Zhuoliang Zan [19,20] each investigated the crack damage of brittle materials and material removal mechanisms using single-grain scratching tests.

Since it is difficult to further improve sawing efficiency and surface quality and there is inevitable surface or subsurface damage, some studies have tried to apply ultrasonic vibration-assisted technology to diamond wire sawing. Liaoyuan Zhang [21] tried to apply ultrasonic vibrations to a diamond wire and found that applying ultrasonic vibrations could improve the sawing efficiency and surface quality. Shujuan Li [22] proposed a method of applying ultrasonic vibrations to a diamond wire along the workpiece feed direction and explored the effects of process parameters and ultrasonic vibration amplitude on sawing efficiency and surface quality through experiments. Lun Li [23] found that the machining trajectory of ultrasonic vibration-assisted sawing showed a fundamental change and that the sawing force of diamond abrasive grains under ultrasonic vibrations become smaller. Yan Wang [24] deduced a sawing force model of a vibration-assisted diamond wire saw, analyzing the impact load generated by the vibration along the feeding direction. SSD (subsurface damage depth) is an important index to evaluate the quality of a machined surface [25]. In order to explore the macroscopic force of a diamond wire saw with vibrations, an ultrasonic vibration-assisted sawing force model with multiple abrasive particles was established [26]. Lutao Yan [27] developed a new 3D vibration-assisted DWS system, established a theoretical prediction model of sawing force, and explored the effects of input vibration direction and sawing parameters on sawing force, surface topography, and tool wear. Sawing temperature can affect the surface quality [28], and the application of ultrasonic vibrations may slightly increase the sawing temperature [29].

It can be seen from the above research that applying vibration assistance can reduce the sawing force and improve both the sawing efficiency and the surface quality of slices. However, diamond wire sawing has gradually transformed from single-wire sawing to multi-wire sawing. The wire mesh formed via multi-wire sawing has an important effect on sawing quality [30]. The original application method of ultrasonic vibration assistance is not easy to achieve, and the influences of other vibration frequencies on diamond wire sawing have not been explored. The parameters of TTV and warp in multi-wire sawing are important factors for measuring surface quality during production [31,32], and it is becoming more and more important to explore the influence of vibration assistance on diamond wire sawing at different frequencies.

Based on the above research, vibration assistance is applied to a working table carrying ingots in the direction of the wire speed. By analyzing the kinematic characteristics, the impact load, and the sawing depth of all the abrasive particles on the wire, an equivalent macroscopic sawing force model is established. A vibration-assisted single-wire-sawing experiment is used to verify the sawing force model and to explore the influences of the sawing parameters and vibration parameters on the sawing force. The influences of vibration assistance on the roughness profile, waviness profile, Ra, and Rz are explored through vibration-assisted single-wire sawing. The influences of vibration assistance on TTV and warp are explored through vibration-assisted multi-wire sawing.

## 2. Materials and Methods

### 2.1. Sawing Force Prediction Model of Vibration-Assisted Sawing

**Figure 1:**
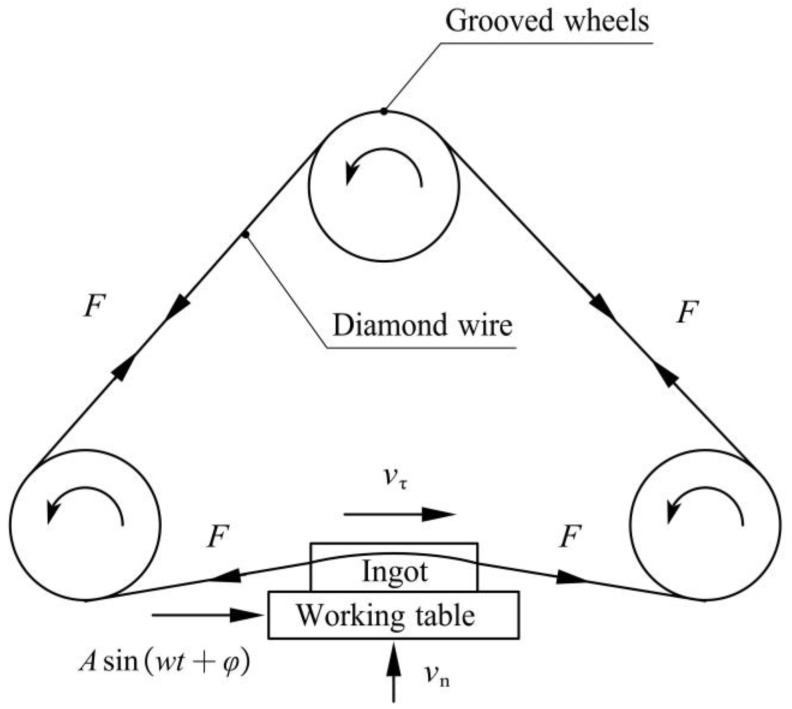
Schematic diagram of diamond wire saw.

The wire mesh of a multi-wire sawing machine was composed of a diamond wire wound sequentially on grooved wheels, and its cross-section was taken to analyze vibration-assisted diamond wire sawing (Figure 1).

Taking the wire speed on the right as an example, $v_{n}$ is the feed speed, $v_{\tau }$ is the wire speed, and F is the wire tension. A certain frequency f and amplitude A are applied to create a simple harmonic vibration along the wire speed to the ingot.

**Figure 2:**
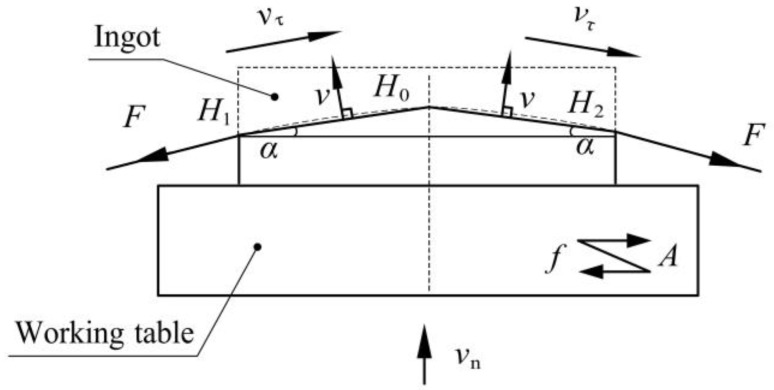
Deformation of the wire saw.

As shown in Figure 2, when analyzing the bending deformation of the wire saw, since the deflection angle of the wire bow α is generally 5–10°, the wire bow can be simplified into two segments of $H_{1}H_{0}$ and $H_{2}H_{0}$. v is the impact of speed on the ingot during vibration.

**Figure 3:**
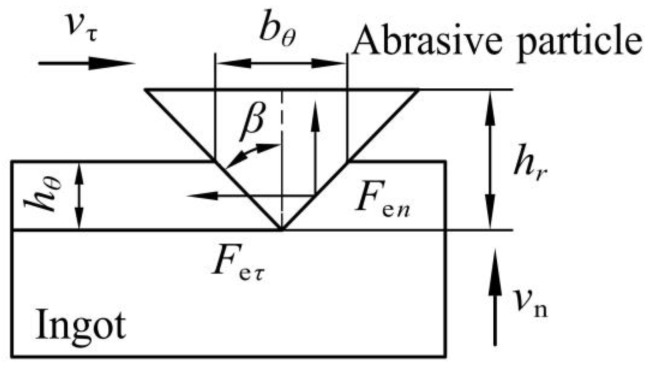
Force model of a single abrasive particle.

Figure 3 shows the force model of a single abrasive particle in wire sawing, where $h_{\theta }$ is the sawing depth of the single abrasive diamond particle, $b_{\theta }$ is the sawing width of the single abrasive diamond particle, and β is the half-cone angle of the abrasive particle.

The sawing forces generated by the sawing ingot are mainly composed of the chip deformation force and friction force. By analyzing the theoretical principle of chip deformation and the tribological theory, the sawing forces $F_{en}$ and $F_{e\tau }$ are determined [14]:

(1) $$\left\{\begin{array}{l} F_{en}=\left(K\tan\beta +\pi \sigma_{sy}\tan^{2}\beta \right)h_{\theta }{}^{2}\\ F_{e\tau }=\left(\frac{K\pi }{4}+\mu \pi \sigma_{sy}\tan^{2}\beta \right)h_{\theta }{}^{2}\\ h_{\theta }=\sqrt{\frac{v_{n}\cos\theta }{Nv_{\tau }\tan\beta }} \end{array}\right.$$

In the formula, K is the coefficient of the chip deformation force (N/mm2), $\sigma_{sy}$ is the average contact pressure strength between the abrasive particle and the ingot (MPa), μ is the friction coefficient between the abrasive particle and the ingot, and N is the number of dynamic abrasive particles per unit area (1/mm^2^).

**Figure 4:**
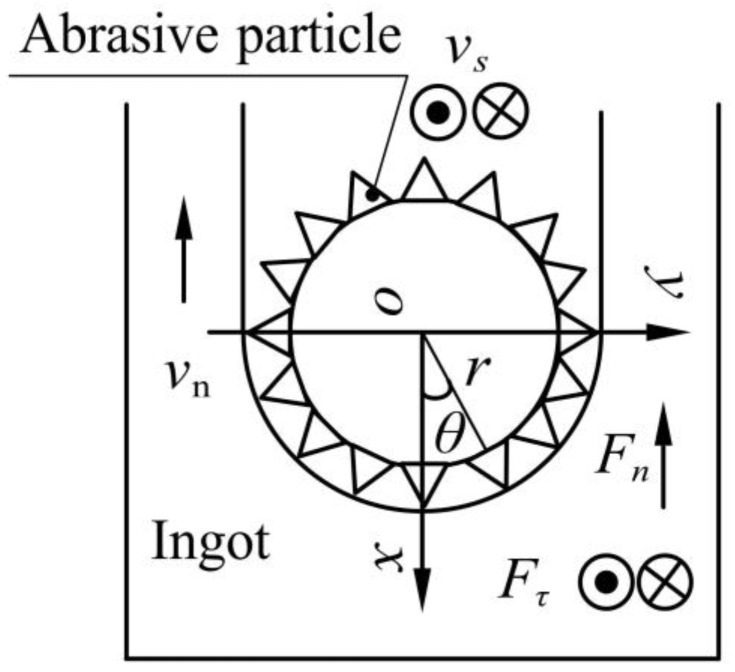
Schematic diagram of the wire cross-section.

Figure 4 shows the force model of the wire cross-section. The normal sawing force $F_{n}$ and tangential sawing force $F_{\tau }$ of all the abrasive particles on the sawing segment can be expressed as follows [14]:

(2) $$\left\{\begin{array}{l} F_{n}=2\int_{0}^{\frac{\pi }{2}}\left(K\tan\beta +\pi \sigma_{sy}\tan^{2}\beta \right)h_{\theta }{}^{2}Nr\cos\theta d\theta l_{s}\\ F_{\tau }=2\int_{0}^{\frac{\pi }{2}}\left(\frac{K\pi }{4}+\mu \pi \sigma_{sy}\tan^{2}\beta \right)h_{\theta }{}^{2}Nrd\theta l_{s} \end{array}\right.$$

In the formula, $l_{s}$ is the sawing length (mm), r is the radius of the wire saw (mm), and θ is the angle of the abrasive particles (°).

**Figure 5:**
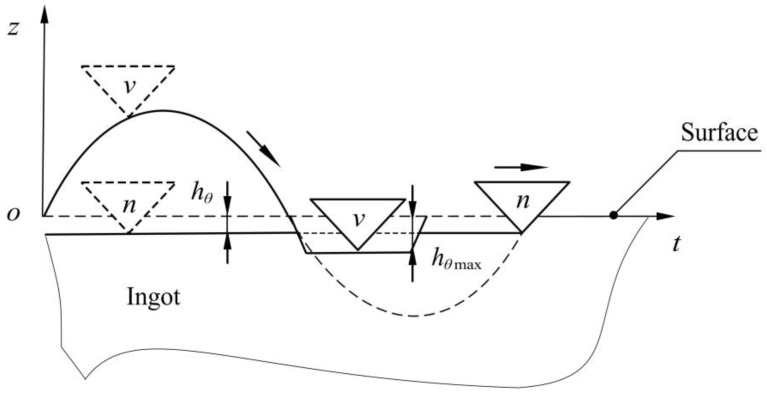
Schematic diagram of the machining trajectory of abrasive particles.

As is shown in Figure 5, when vibration is applied, the trajectory of the abrasive particles changes, the abrasive grains contact the workpiece intermittently, and the trajectory of the abrasive particles approximates a sine curve. Since the feed speed is much smaller than the vibration speed, it can be calculated that the time it takes for abrasive particles to participate in sawing within one vibration period is T/4, where T is a single vibration period.

When analyzing the vibration impact, the maximum sawing depth $h_{\theta \max}$ and the normal impact load $F_{\mathrm{env}}$ of the abrasive particles are determined as follows:

(3) $$\left\{\begin{array}{l} h_{\theta \max}={\left(\frac{3Mv{\left(\frac{T}{2}\right)}^{2}}{2\left(K\tan\beta +\pi \sigma_{sy}\tan^{2}\beta \right)}\right)}^{\frac{1}{3}}\\ F_{\mathrm{env}}=\left(K\tan\beta +\pi \sigma_{sy}\tan^{2}\beta \right)h_{\theta \max}{}^{2} \end{array}\right.$$

In the formula, M is the equivalent mass of the wire saw (kg), and v is the normal speed of the abrasive particles under impact (mm/s).

According to the principle of equal volume, the equivalent action time of a single abrasive particle in one vibration period $t_{\theta }$ can be determined:

(4) $$t_{\theta }=\frac{T{\left(F_{en}\right)}^{\frac{9}{8}}}{{\left(F_{env}\right)}^{\frac{9}{8}}}$$

According to the impulse theorem, the average normal sawing force and the average tangential sawing force of a single abrasive particle in one vibration period can be expressed as follows:

(5) $$\left\{\begin{array}{l} F_{nva}=\frac{I}{T}=\frac{F_{en}t_{\theta }}{T}=\frac{\left(K\tan\beta +\pi \sigma_{sy}\tan^{2}\beta \right)h_{\theta \max}{}^{2}t_{\theta }}{T}\\ F_{\tau va}=\frac{I}{T}=\frac{F_{e\tau }t_{\theta }}{T}=\frac{\left(\frac{K\pi }{4}+\mu \pi \sigma_{sy}\tan^{2}\beta \right)h_{\theta \max}{}^{2}t_{\theta }}{T} \end{array}\right.$$

With reference to Formula (2) for sawing forces in conventional sawing, the following formula determines the sawing forces in vibration-assisted wire sawing:

(6) $$\left\{\begin{array}{l} F_{nv}=2\int_{0}^{\frac{\pi }{2}}\frac{\left(K\tan\beta +\pi \sigma_{sy}\tan^{2}\beta \right)h_{\theta \max}{}^{2}t_{\theta }}{T}Nr\cos\theta d\theta l_{s}\\ F_{\tau v}=2\int_{0}^{\frac{\pi }{2}}\frac{\left(\frac{K\pi }{4}+\mu \pi \sigma_{sy}\tan^{2}\beta \right)h_{\theta \max}{}^{2}t_{\theta }}{T}Nrd\theta l_{s} \end{array}\right.$$

### 2.2. Vibration-Assisted Diamond-Wire-Sawing Experiment

#### 2.2.1. Design of Experimental Device

**Figure 6:**
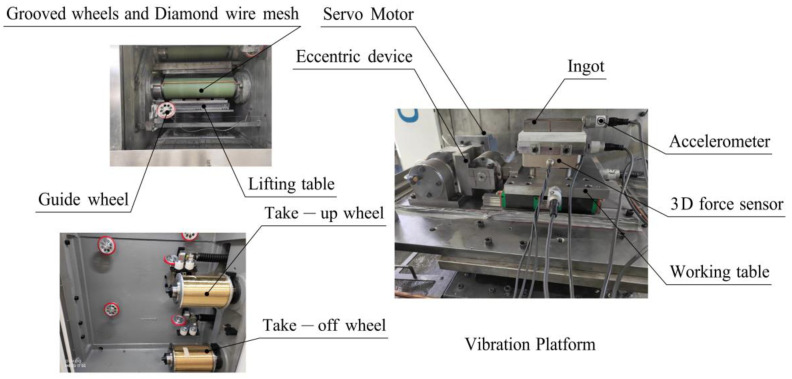
The structure of the experimental machine tool.

As shown in Figure 6, the vibration-assisted sawing experiment was carried out on a DX-2260 multi-wire sawing machine tool with a vibration-assisted platform. The ingot was fixed to the platform, and the lifting table exhibited a top–down feed movement. The diamond wire saw was wound on the grooved wheels, the retracting wheels, and the guide wheels of the machine tool. Diamond wire sawing was achieved through the feed movement of the lifting platform and the reciprocating motion of the wire saw.

The vibration-assisted platform consisted of three parts: the servo motor, the eccentric device, and the working table. The eccentric device converted the rotation of the servo motor to the vibration of the working table along the wire speed. The motion of the vibration platform and ingot could be monitored by the accelerometer. Through the control system of the vibration-assisted machine tool, the selection and adjustment of vibration parameters and sawing parameters could be realized.

The ranges of the vibration parameters, sawing parameters, and ingot size in the experiment equipment are shown in Table 1. The wire length could be changed between 100 and 430 mm. In order to ensure surface quality and sawing efficiency, the wire speed was adjusted between 0 and 20 m/s, the feed speed was below 60 mm/h, the wire radius was between 0.05 and 0.15 mm, the wire tension was between 20 and 40 N, and the vibration frequency was between 0 and 50 Hz.

#### 2.2.2. Experimental Measurements

**Figure 7:**
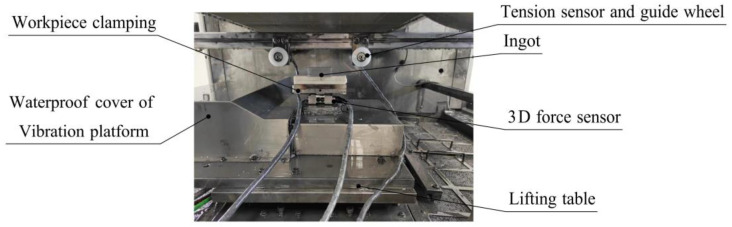
Measurement of experimental parameters.

To verify the vibration equivalent sawing force model, it was necessary to obtain the $F_{nv}$ and $F_{\tau v}$ values corresponding to different sawing parameters via a vibration-assisted single-wire-sawing experiment and to compare them with $F_{n}$ and $F_{\tau }$ values. The measurement of the sawing forces in the vibration-assisted single-wire experiments is shown in Figure 7. A 3D force sensor was mounted on a worktable that carried the ingot, then the sawing force between the diamond wire and ingot, $F_{nv}$ and $F_{\tau v}$, respectively, was measured. A tension sensor was installed on the two guide wheels, which were symmetrically distributed above the ingot, and was used to measure the wire tension F on two sides.

We used the same batch of HT250, stainless steel, NdFeB, and SiC as experimental ingots after aging treatment, and the ingots were square. In the single-wire-sawing experiment, the surface roughness and surface profiles were scanned and measured using a Bruker Dektak XT instrument to obtain the Ra, Rz, roughness profile, waviness profile, and thickness profile. In the multi-wire-sawing experiment, the TTV and warp of the slices were measured to evaluate the average surface quality.

## 3. Results and Discussion

### 3.1. Vibration Equivalent Sawing Force Analysis

According to the adjustable value range of the experimental equipment, the same batch of HT250 was selected to ensure that the material properties were essentially the same. The orthogonal method was used to explore the influences of vibration frequency, feed speed, wire speed, and tension on the tangential sawing force and normal sawing force in vibration-assisted single-wire sawing. To verify the accuracy of the vibration equivalent sawing force model, we selected certain vibration parameters and sawing parameters (Table 2).

The experiments were carried out according to the method described in Table 2. We input the sawing parameters into the vibration equivalent sawing force model to calculate the corresponding $F_{nv}$ and $F_{\tau v}$ values. Repeated experiments were carried out, and the average values and standard deviations of the experimental values $F_{nvc}$ and $F_{\tau vc}$ were obtained. By comparing the theoretical values of $F_{nv}$ and $F_{\tau v}$ with the experimental values of $F_{nvc}$ and $F_{\tau vc}$, the accuracy of the sawing force model was verified. The influences of the sawing parameters and vibration parameters on the tangential sawing force and normal sawing force were determined, and the influence of the sawing parameters on vibration assistance was explored.

**Figure 8:**
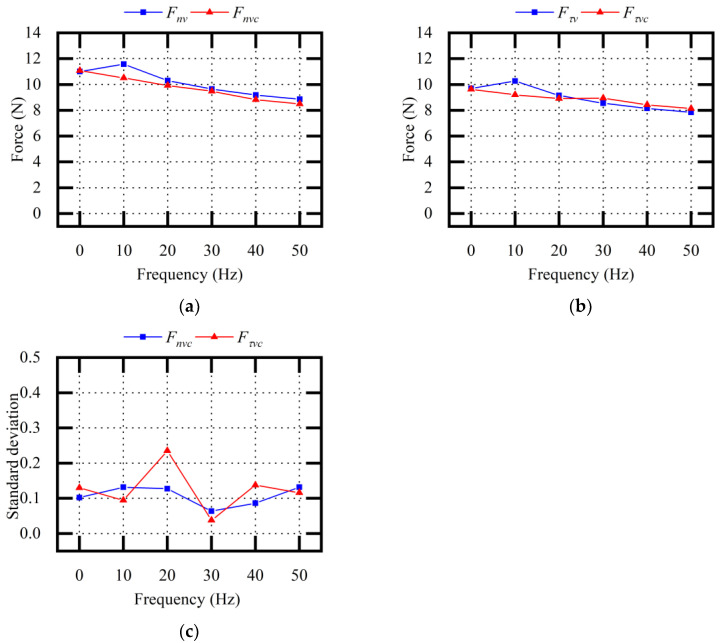
Comparative analysis of sawing forces (variable f): (**a**) normal sawing force; (**b**) tangential sawing force; (**c**) standard deviation σ_1_ of experimental values.

Figure 8 shows the theoretical values and the experimental values when changing the vibration parameters f. In vibration-assisted wire sawing, both $F_{nv}$ and $F_{\tau v}$ decrease by about 20% at 50 Hz, and the effect of vibration assistance was enhanced with the increase in vibration frequency. The average relative error between the theoretical $F_{nv}$ value and the experimental $F_{nvc}$ value was 4.66%. The average relative error between the theoretical $F_{\tau v}$ value and the experimental $F_{\tau vc}$ value was 4.82%.

Through the description of the above experimental results, it was concluded that vibration assistance could effectively reduce the sawing forces during sawing and that vibration frequency was one of the important parameters affecting the sawing force.

**Figure 9:**
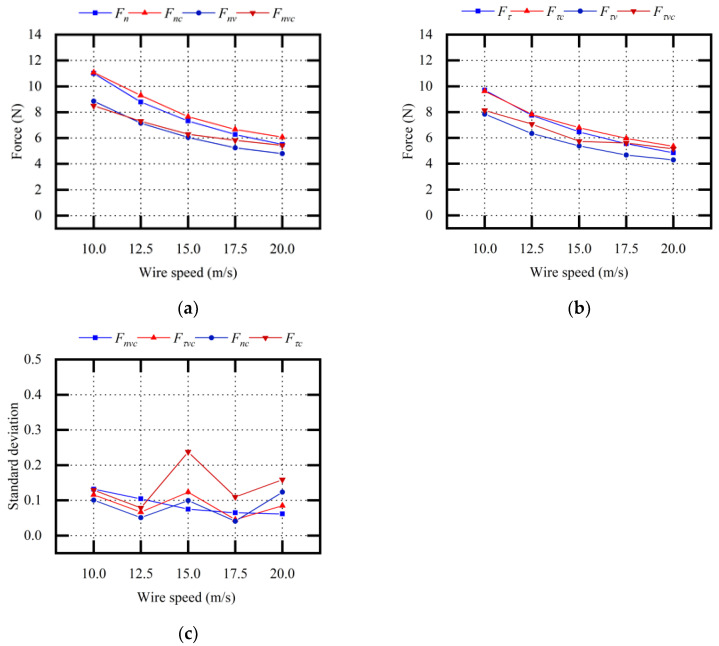
Comparative analysis of sawing force (variable $v_{\tau }$): (**a**) normal sawing force; (**b**) tangential sawing force; (**c**) standard deviation σ_1_ of experimental values.

Figure 9 shows the theoretical values and experimental values when changing the sawing parameters $v_{\tau }$. With or without vibration assistance, the sawing forces gradually decreased with the increase in $v_{\tau }$. The sawing forces $F_{nv}$ and $F_{\tau v}$ in vibration-assisted wire sawing were smaller than the sawing forces $F_{n}$ and $F_{\tau }$ in conventional wire sawing. The average relative error between the theoretical $F_{nv}$ value and the experimental $F_{nvc}$ value was 6.34%. The average relative error between the theoretical $F_{\tau v}$ value and the experimental $F_{\tau vc}$ value was 10.63%.

It was predicted that, with an increase in $v_{\tau }$, the sawing force would decrease, the wire bow deflection and deflection angle would decrease, and the impact load between the abrasive particle and the ingot would be weakened. Therefore, the optimization effect of vibration assistance on sawing force was relatively weak. Through the description of the above experimental results and the force model, it was concluded that the wire speed affected the effect of vibration assistance on the sawing force. The effect of vibration assistance was slightly weakened with the increase in wire speed.

**Figure 10:**
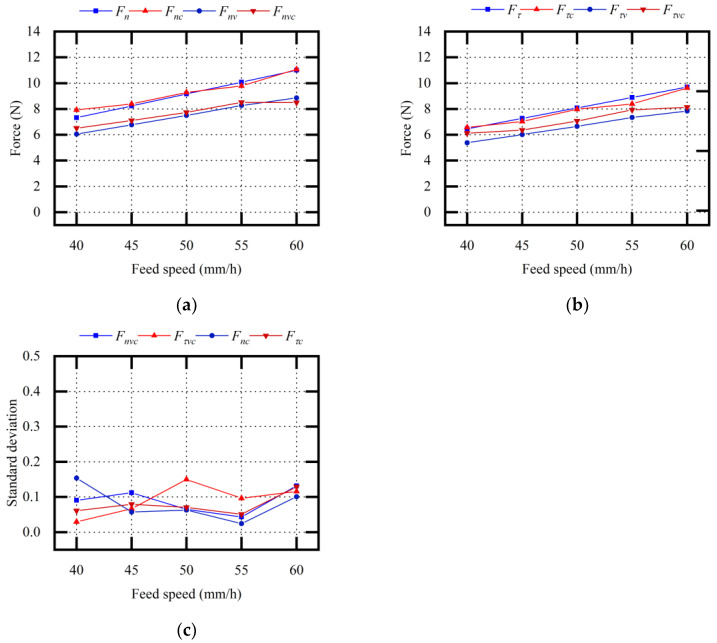
Comparative analysis of sawing force (variable $v_{n}$): (**a**) normal sawing force; (**b**) tangential sawing force; (**c**) standard deviation σ_1_ of experimental values.

Figure 10 shows the theoretical values and experimental values when changing the sawing parameters $v_{n}$. With or without vibration assistance, the sawing forces gradually increased with the increase in $v_{\tau }$. $F_{nv}$ and $F_{\tau v}$ were smaller than $F_{n}$ and $F_{\tau }$. The average relative error between the theoretical $F_{nv}$ value and the experimental $F_{nvc}$ value was 4.32%. The average relative error between the theoretical $F_{\tau v}$ value and the experimental $F_{\tau vc}$ value was 6.88%.

It was predicted that, with an increase in $v_{n}$, the sawing force would increase, the wire bow deflection and deflection angle would increase, and the impact load between the abrasive particle and the ingot would be weakened. Therefore, the optimization effect of vibration assistance on sawing force was a slight enhancement. Through the description of the above experimental results and the force model, it was concluded that feed speed affected the effect of vibration assistance on the sawing force. The effect of vibration assistance was slightly enhanced with the increase in feed speed.

**Figure 11:**
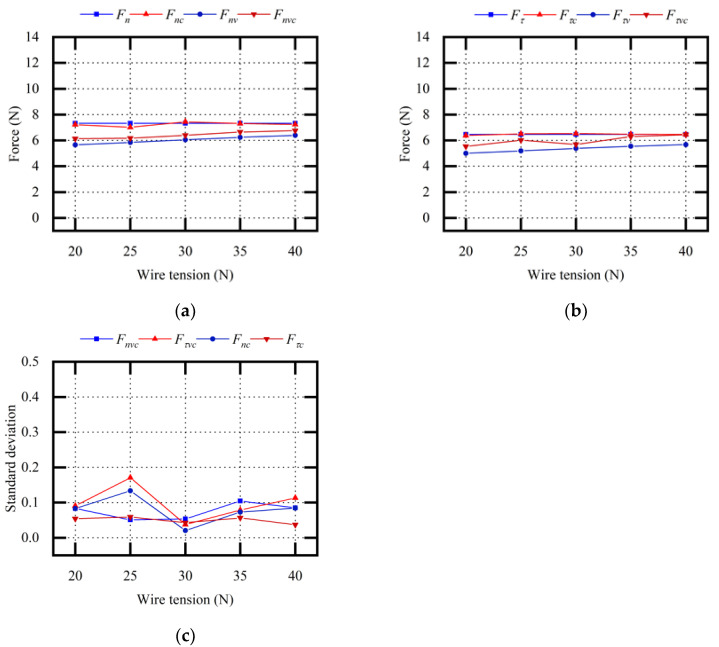
Comparative analysis of sawing force (variable F): (**a**) normal sawing force; (**b**) tangential sawing force; (**c**) standard deviation σ_1_ of experimental values.

Figure 11 shows the theoretical calculated values and experimental measured data when changing the sawing parameters F. When vibration assistance was not applied, $F_{n}$ and $F_{\tau }$ were not affected by the wire tension F. When vibration assistance was applied, $F_{nv}$ and $F_{\tau v}$ gradually increased with the increase in wire tension F. $F_{nv}$ and $F_{\tau v}$ were smaller than $F_{n}$ and $F_{\tau }$. The average relative error between the theoretical $F_{nv}$ value and the experimental $F_{nvc}$ value was 5.96%. The average relative error between the theoretical $F_{\tau v}$ value and the experimental $F_{\tau vc}$ value was 10.59%.

It was predicted that, with an increase in F, the wire bow deflection and deflection angle would decrease, and the impact load between the abrasive particle and ingot would be weakened. Therefore, the optimization effect of vibration assistance on sawing force was relatively weak. Through the description of the above experimental results and the force model, it was concluded that wire tension affected the effect of vibration assistance on the sawing force. The effect of vibration assistance was slightly reduced with the increase in wire tension.

An orthogonal experiment method was adopted, and the following discussion results were arrived at by comparing the experimental results with the theoretical results:

- The relative error between the experimental values and the theoretical values was relatively small, which verified the accuracy of the sawing force prediction model;
- With increase in the vibration frequency (0–50 Hz), the sawing forces $F_{nv}$ and $F_{\tau v}$ gradually decreased;
- Sawing parameters, including wire speed, feed speed, and wire tension, affected the impact load between the abrasive particle and the ingot and further influenced the optimization effect of vibration assistance on the sawing forces $F_{nv}$ and $F_{\tau v}$.

### 3.2. Study on the Surface Quality of Vibration-Assisted Sawing

HT250, NdFeB, stainless steel, and other materials were selected to explore the influence of vibration assistance on the roughness profiles, Ra and Rz values, waviness profiles, and thickness profiles of the slices. The change trend in the waviness profile was consistent with that in the thickness profile, which reflected the quality difference on the same surface. Ra and Rz at different positions on the same surface were scanned and measured, and the average values and standard deviations of Ra and Rz were obtained. The selected vibration parameters and sawing parameters are shown in Table 3.

The experiments were carried out according to the method described in Table 3. We obtained the roughness profiles, the waviness profiles, and the Ra and Rz values corresponding to the scanning position of the slices and explored the influence of vibration assistance on surface quality.

**Figure 12:**
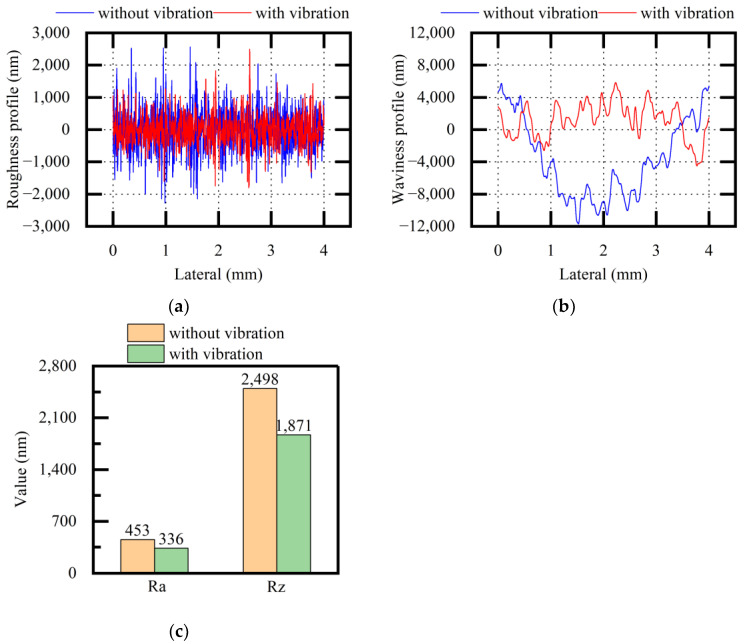
Comparative analysis of surface quality (HT250, variable f): (**a**) roughness profile; (**b**) waviness profile; (**c**) average values of Ra and Rz.

Figure 12 shows the experimental results of the roughness profiles, waviness profiles, and Ra and Rz values of the HT250 surfaces (both when applying vibration and when not applying vibration). The roughness profiles and waviness profiles became relatively stable, and the quality difference in terms of HT250 surfaces was reduced when applying vibration. The roughness values of Ra and Rz were also reduced when applying vibration. The standard deviations of Ra were 11.20 and 7.83 without and with vibration, respectively. The standard deviations of Rz were 33.74 and 27.59 without and with vibration, respectively. Through the description of the above experimental results, it was concluded that vibration assistance could optimize the surface quality factors to different degrees when sawing HT250.

**Figure 13:**
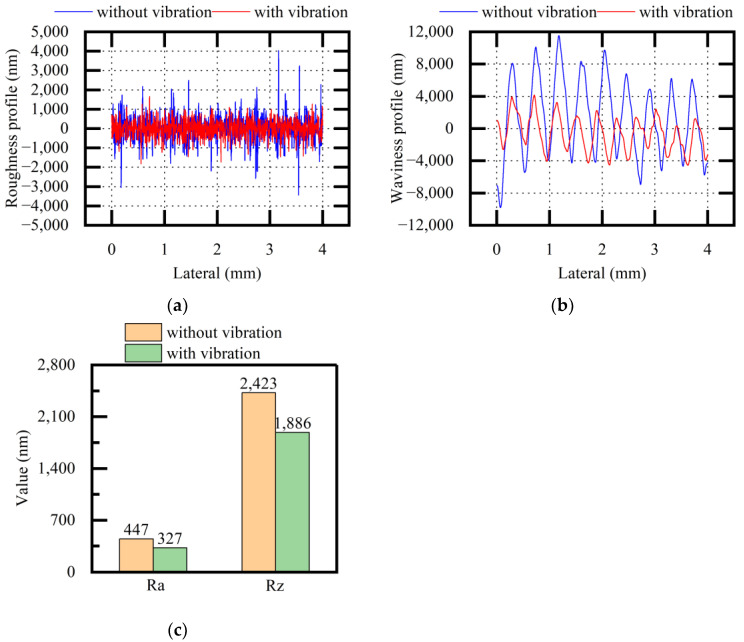
Comparative analysis of surface quality (stainless steel, variable f): (**a**) roughness profile; (**b**) waviness profile; (**c**) average values of Ra and Rz.

Figure 13 shows the experimental results of the roughness profiles, waviness profiles, and Ra and Rz values of the stainless-steel surfaces (both when applying vibration and when not applying vibration). After vibration assistance was applied, the measured values showed that the roughness profiles and the waviness profiles became relatively stable, and the quality difference on the same surface was significantly reduced. The roughness values of Ra and Rz were also reduced when applying vibration. The standard deviations of Ra were 11.86 and 16.54 without and with vibration, respectively. The standard deviations of Rz were 31.90 and 99.33 without and with vibration, respectively. Through the description of the above experimental results, it was concluded that vibration assistance could optimize the surface quality factors to different degrees when sawing stainless steel.

**Figure 14:**
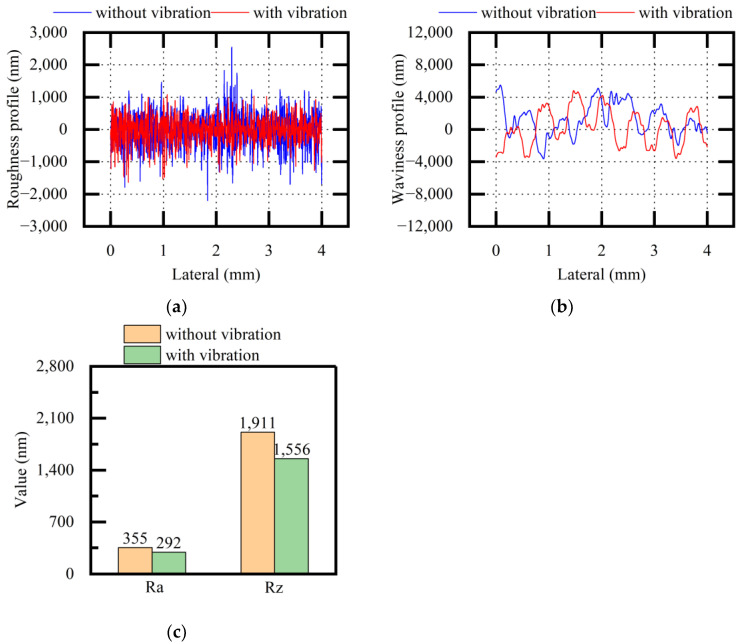
Comparative analysis of surface quality (NdFeB, variable f): (**a**) roughness profile; (**b**) waviness profile; (**c**) average values of Ra and Rz.

Figure 14 shows the experimental results of the roughness profiles, waviness profiles, and Ra and Rz values of the NdFeB surfaces (both when applying vibration and when not applying vibration). After vibration assistance was applied, the measured values showed that the roughness values of the surface decreased; however, compared with the waviness profiles of the HT250 surfaces and stainless-steel surfaces, the quality difference on the same NdFeB surface was smaller, and a difference in terms of the waviness profile was not obvious from the measured values. The standard deviations of Ra were 6.78 and 7.88 without and with vibration, respectively. The standard deviations of Rz were 132.72 and 27.36 without and with vibration, respectively. Through the description of the above experimental results, it was inferred that the optimization effect of vibration assistance was more significant when the original quality difference on the same surface was large during conventional sawing.

Based on the comparative experimental results, the following discussion results could be drawn:

- The abrasive particles produced a polishing effect on the surfaces during vibration-assisted sawing;
- Vibration assistance could reduce the surface roughness values of Ra and Rz for different materials;
- The worse the stability of the surface quality in conventional sawing, the more significant the optimization effect of vibration assistance on quality difference.

### 3.3. Experimental Results of Vibration-Assisted Multi-Wire Sawing

The single-wire-sawing experiment was extended to a multi-wire-sawing experiment, and the geometric parameters of slice TTV and warp values with different vibration parameters and sawing parameters were measured to explore the influence of vibration assistance on average surface quality. The average values, maximum values, and standard deviations σ_3_ of TTV and warp were obtained. The vibration parameters and sawing parameters are shown in Table 4.

According to the experimental method shown in Table 4, the vibration-assisted multi-wire-sawing experiment was carried out to obtain the TTV and warp values and to explore the influence of vibration on TTV, warp, and other surface quality parameters in multi-wire sawing.

**Figure 15:**
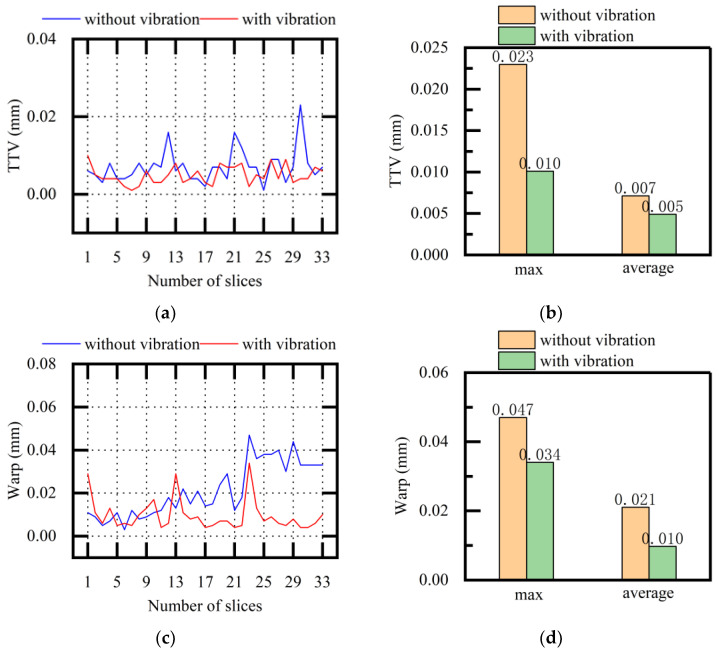
Comparative analysis of TTV and warp (NdFeB, variable f): (**a**) TTV of each slice; (**b**) maximum and average values of TTV; (**c**) warp of each slice; (**d**) maximum and average warp values.

In Figure 15, the experimental TTV and warp values of NdFeB slices obtained when using multi-wire sawing (both with and without vibration) are compared. After vibration assistance was applied, the standard deviations of TTV were 0.0043 and 0.0023 without and with vibration, respectively. The standard deviations of warp were 0.0124 and 0.0074 without and with vibration, respectively. The average values, maximum values, and standard deviations of both TTV and warp were reduced. Through the description of the above experimental results, it was concluded that the overall surface quality of the slices tended to be stable and that the sawing stability was improved when using multi-wire sawing on NdFeB.

**Figure 16:**
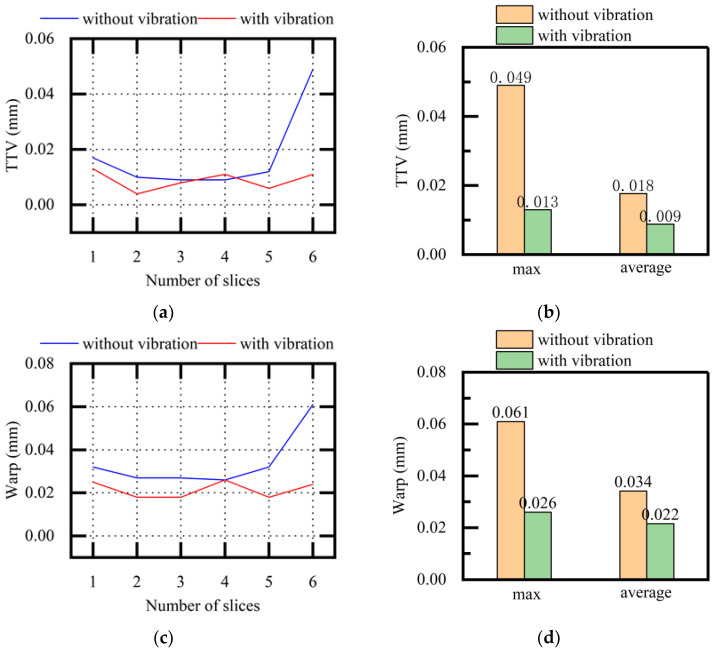
Comparative analysis of TTV and warp (SiC, variable f): (**a**) TTV of each slice; (**b**) maximum and average TTV; (**c**) warp of each slice; (**d**) maximum and average warp.

In Figure 16, the experimental TTV and warp values of SiC slices obtained when using multi-wire sawing (both with and without vibration) are compared. After vibration assistance was applied, the standard deviations of TTV were 0.0143 and 0.0031 without and with vibration, respectively. The standard deviations of warp were 0.0122 and 0.0035 without and with vibration, respectively. The average values, maximum values, and standard deviations of both TTV and warp were reduced. Through the description of the above experimental results, it was concluded that the overall surface quality of the slices tended to be stable and that the sawing stability was improved when using multi-wire sawing on SiC.

TTV and warp are important standards for measuring the quality of slices. In order to meet the needs of large-sized, high-precision, multi-slice processing, it is necessary to obtain good surface precision with good TTV and warp values. An unqualified TTV affects the chip pass rate and the production process, and the evaluation standards also put forward strict requirements for chip quality. Based on the above results, we concluded that the average values, maximum values, and standard deviations of TTV and warp were relatively reduced and that the sawing stability and surface quality were improved when using multi-wire sawing. Vibration-assisted technology could improve the pass rate of slices and optimize the production process.

## 4. Conclusions

In this paper, a method of applying vibration assistance to ingot slicing was presented that attempted to extend vibration assistance to the frequency range of 0–50 Hz. A sawing force prediction model for vibration-assisted wire sawing was established. The prediction model described the theoretical relationship between the process parameters and sawing forces. The relationships between sawing forces and process parameters, such as vibration frequency, wire speed, feed speed, and wire tension, were explored through experiments. The effects of vibration assistance on surface quality were explored in both single-wire-sawing and multi-wire-sawing contexts.

The conclusions that could be drawn based on the results were as follows:

- In wire sawing, the deformation of the wire saw and the vibration assistance affected each other;
- During vibration-assisted wire sawing, the sawing forces could be reduced by different sawing parameters;
- Vibration frequency, feed speed, wire speed, and wire tension were important factors that affected the sawing force, and the effect of vibration assistance on the sawing force was comprehensively affected by the sawing parameters and vibration parameters;
- The surface roughness, quality difference on the same surface, TTV, and warp were all reduced during vibration-assisted wire sawing;
- It was predicted that the polishing effect of abrasive particles on the surface would be enhanced and that chip removal would be improved when using vibration-assisted sawing;
- The selection and matching of materials, vibration parameters, and sawing parameters were based on the prediction force model and the experiments described in this paper.

## Figures and Tables

**Table 1:** Adjustable ranges of experimental parameters.

f/Hz	$v_{n}$/mm/h	$v_{\tau }$/m/s	F/N	L/mm	l/mm	r/mm
0–50	0–60	0–20	20–40	100–430	0–180	0.05–0.15

**Table 2:** Experimental design of sawing force analysis.

Material	Variable	f/Hz	$v_{\tau }$/m/s	$v_{n}$/mm/h	F/N	l/mm	L/mm
HT250	Frequency f	0	10.0	60	30	120	200
		10					
		20					
		30					
		40					
		50					
HT250	Wire speed $v_{\tau }$	50	10.0	60	30	120	200
			12.5				
			15.0				
			17.5				
			20.0				
HT250	Feed speed $v_{n}$	50	10.0	40	30	120	200
				45			
				50			
				55			
				60			
HT250	Wire tension F	50	15.0	60	20	120	200
					25		
					30		
					35		
					40		

**Table 3:** Experimental design of surface quality.

Material	Variable	f/Hz	$v_{\tau }$/m/s	$v_{n}$/mm/h	F/N	l/mm	L/mm
HT250	Frequency f	0	10.0	60	30	120	200
		50					
Stainless steel	Frequency f	0	10.0	60	30	120	200
		50					
NdFeB	Frequency f	0	10.0	60	30	100	200
		50					

**Table 4:** Experimental design of vibration-assisted multi-wire sawing.

Material	Variable	f/Hz	$v_{\tau }$/m/s	$v_{n}$/mm/h	F/N	l/mm	L/mm
NdFeB	Frequency f	0	15.0	40	30	100	430
		50					
SiC	Frequency f	0	20.0	8	35	22	430
		50					

## Data Availability

Not applicable.
